# Vitamin D3 encapsulated in polymeric nanoparticles to dampen the pro-inflammatory immune response

**DOI:** 10.1016/j.jtauto.2025.100321

**Published:** 2025-09-30

**Authors:** Julia Minnee, Jorge Cuenca-Escalona, Johanna Bödder, Georgina Flórez-Grau, Esther C. de Jong, I. Jolanda M. de Vries

**Affiliations:** a Department of Medical BioSciences, Radboud University Medical Center, Nijmegen, the Netherlands; b Department Experimental Immunology, Amsterdam UMC, location AMC, Amsterdam Institute for Infection and Immunity, Amsterdam, the Netherlands

**Keywords:** Anti-inflammatory, Nanoparticle, Phagocyte, Suppression, Therapy, Tolerance, Vitamin D3

## Abstract

1α25-dihydroxyvitamin D3, the active metabolite of vitamin D3 (VD3), is a modulator of inflammation well-known for its ability to promote anti-inflammatory and tolerogenic immune responses. It is therefore an attractive agent for the attenuation of inflammatory responses and the development of tolerogenic immunity in autoimmune diseases. To overcome VD3 toxicity and enhance its *in vivo* performance, nanoparticles (NPs) have emerged as a promising delivery platform. Therefore, in this study, we have developed VD3-loaded polymeric nanoparticles (VD3-NPs) as a therapeutical strategy for the treatment of autoimmune disorders. We demonstrate that VD3-NPs could successfully be generated and that they significantly inhibit secretion of IL-6, IL-10, IL-23, and TNFα in human whole blood cultures. We observed that poly(lactic-co-glycolic acid) (PLGA) NPs are efficiently taken up by neutrophils, monocytes and B cells, prompting further investigation into the effect of VD3-NPs on these subsets. Investigation into each of the immune cell subsets demonstrated that the VD3-NPs were able inhibit cytokine secretion by both monocytes and neutrophils. Moreover, VD3-NPs induced a tolerogenic phenotype in monocytes. In B cells, we observed that VD3-NPs impaired *in vitro* plasma B cell differentiation and suppressed antibody production. Together, our results validate for the first time in primary human cells the therapeutic potential of VD3 encapsulated in PLGA NPs, posing an attractive strategy for the treatment of autoimmune diseases.

## Graphical abstract

**Figure:**
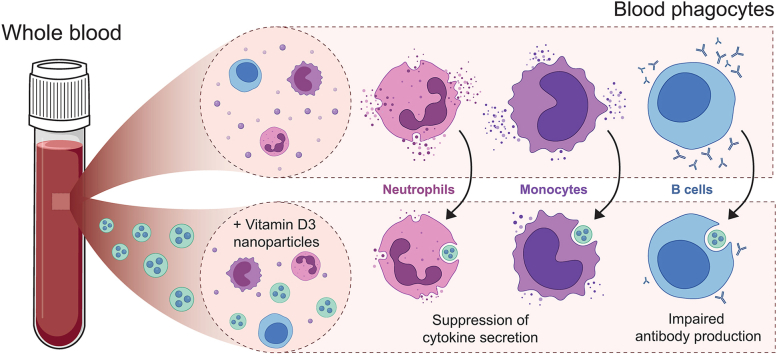

## 1 Introduction

Autoimmune diseases encompass a diverse range of disorders characterized by a loss of tolerance, resulting in inflammation and chronic immune-mediated damage to healthy tissues [1]. The pathogenesis of autoimmune diseases is often not fully understood, however they commonly involve the activation of autoreactive T and B cells, elevated levels of autoantibodies and an abundance of pro-inflammatory cytokines such as type I interferons, tumor necrosis factor (TNF), and proinflammatory interleukins (IL) [[2], [3], [4], [5], [6]]. Despite significant advances in the treatment of autoimmune diseases, current therapies comprise of chronic drug regimens for symptom management while failing to achieve curative long-term effects in patients [[7], [8], [9], [10]]. Thus, there remains a critical need for the development of new therapeutic strategies to target the underlying immunological processes that drive the onset and progression of autoimmune diseases, with the goal of reducing disease activity, minimizing the need for long-term medication, and ultimately achieving disease remission, prevention, or cure.

The active metabolite of vitamin D3 (VD3), 1α25-dihydroxyvitamin D3, is a well-known modulator of inflammation that exerts its effects through the vitamin D receptor (VDR), expressed in multiple immune cell subsets [11]. Through VDR signalling, VD3 promotes anti-inflammatory and tolerogenic responses by regulating key genes involved in immune cell differentiation, maturation, metabolism, and responsiveness to cytokines and chemokines [12,13]. Due to these immunomodulatory properties, VD3 is regarded as an attractive therapeutic agent for the attenuation of proinflammatory immunity and favouring the development of tolerogenic immunity in autoimmune diseases. Unfortunately, the active VD3 metabolite is inherently unstable, hence displaying suboptimal functionalities *in vivo* in addition to exhibiting acute toxicities at high dosages [14]. Therefore, it is advantageous to develop alternative delivery strategies to boost the therapeutic performance of VD3 *in vivo*. In this regard, nanoparticles (NPs) have emerged as a promising drug delivery platform, offering advantages over the use of soluble drugs such as sustained release, protection of the bioactive compound from degradation, and targeted delivery to phagocytic immune cell populations. Poly(D,L-lactic-co-glycolic acid) (PLGA) stands out as a biodegradable and biocompatible polymer and is widely used for the development of NP-based drug delivery systems [[15], [16], [17]].

While the immunomodulatory effect of VD3 has been extensively studied, whether VD3 encapsulated in PLGA NPs poses a therapeutic strategy in humans remains to be elucidated [[18], [19], [20], [21]]. Therefore, this study represents a first proof-of-concept for the development of immunosuppressive VD3-loaded polymeric NPs (VD3-NPs) for their clinical application as treatment of autoimmune disorders in humans. Aiming to mimic the application of these NPs through intravenous (IV) injection, we demonstrate that VD3-NPs significantly inhibit cytokine secretion in human whole blood cultures. We observed that PLGA NPs are efficiently taken up by neutrophils, monocytes, and B cells amongst the prominently present immune cell subsets in the blood, prompting further investigation into the effect of VD3-NPs on these cell types. Investigation into each of the immune cell subsets separately demonstrated that the VD3-NPs were able to successfully attenuate inflammation and steer the immune response towards an anti-inflammatory state. Together, our results demonstrate the therapeutic potential of VD3 encapsulated in PLGA NPs, proven by their capacity to impair the development of pro-inflammatory immunity, therefore posing an attractive strategy for the treatment of autoimmune diseases.

## 2 Methods

### 2.1 Nanoparticle synthesis

The protocol for NP synthesis was adapted from Bödder et al. [22]. Briefly, PLGA NPs containing VD3 or dye were prepared using a single emulsion and solvent evaporation-extraction method. 100 mg of PLGA 50:50 Resomer 502H (26780-50-7, Evonik Industries) was dissolved in 3 mL dichloromethane (1.06049, Merck) which was used as organic solvent. For the synthesis of VD3-NPs, dye-NPs and dye-VD3-NPs, calcitriol (32222-06-3, CARBOGEN AMCIS) and/or ATTO Oxa12 carboxy (AD Oxa12-25, Atto TEC) dye dissolved in dimethyl sulfoxide (DMSO) (472301, Sigma Aldrich) was added to the organic solvent to obtain 1 wt% VD3 and/or 0.05 wt% dye in ratio to the weight of PLGA added. For the synthesis of empty NPs, nothing was added to the organic solvent. The organic solvent was added dropwise to a 25 mL solution of 2.5 % poly-vinyl alcohol (360627, Sigma Aldrich) while under sonication (2 × 58 s at 20 % amplitude). Upon overnight evaporation of the solvent at 4 °C, the NPs were collected, washed with MilliQ and centrifuged at 11000 rpm for 20 min at 4 °C for a total of 3 washes. The NPs were then dissolved in MilliQ, lyophilized, and stored at −20 °C until further use.

### 2.2 Nanoparticle characterization

The NPs were characterized for average size and polydispersity index (PDI) via dynamic light scattering (DLS). For DLS analysis, lyophilized NPs were dissolved in MilliQ at 0.5 mg/mL before measuring on a NANO-flex (Microtrac Inc) and analysed using Microtrac FLEX Application Software (Version 11.1.0.2, Microtrac Inc). VD3 encapsulation was determined using high-performance liquid chromatography (HPLC) (Shimadzu Corporations). For HPLC analysis, lyophilized NPs were dissolved in DMSO and further diluted in 1:1 acetonitrile (83639.320, VWR Chemicals) and water for injection (362 4315, B. Braun) with 0.05 % trifluoroacetic acid (T0699, Merck) to an end concentration of 5 mg/mL. The dissolved NPs were centrifuged at 10000 rpm for 10 min at 4 °C after which 25 μL was of the supernatant was injected into the HPLC using an XSelect Peptide CSH C18 Column (4.6 mm × 100 mm, 3.5 μm particle size) (186006956, Waters). The output was analysed using LabSolutions software (Version 5.111, Shimadzu Corporation). The concentration of VD3 was determined based on a linear standard curve generated with the VD3 standards that were measured on the same day. The final wt% of VD3 was calculated as follows: (calculated weight of VD3 measured after synthesis/total weight of NPs after synthesis) ∗ 100. The NPs were tested for the presence of endotoxin using the ToxinSensor Chromogenic LAL Endotoxin Assay Kit (L00350, GenScript) according to manufacturer's protocol.

### 2.3 Whole blood cytokine secretion

Heparin-treated blood from healthy donors (Bloodbank Sanquin, Nijmegen) was diluted 1:1 in X-VIVO medium (02-053Q, Lonza) and plated at 200 μL per well in a 96-well U bottom plate. The blood samples were either left unstimulated or treated with soluble VD3 or VD3-NP with a final VD3 concentration of 0.1 μM. For empty NP treatment, the concentration of NPs added was adjusted to the concentration of the VD3-NPs. After overnight incubation at 37 °C, lipopolysaccharide (LPS) (100 ng/mL) (vac-3pelps, InvivoGen) or medium was added to the cultures followed by 6 h of incubation at 37 °C. The culture plate was centrifuged at 1500 rpm for 5 min at 4 °C after which the supernatant was harvested and stored at −80 °C until further analysis. The samples were resuspended in 100 μL ammonium-chloride-potassium (ACK) lysis buffer for 10 min at room temperature (RT) for removal of erythrocytes, followed by a wash with PBS. Viability of CD45^+^ cells was then analysed by flow cytometry.

### 2.4 Uptake of NPs in whole blood

Heparin-treated blood from healthy donors (Bloodbank Sanquin, Nijmegen) was diluted in PBS to get a concentration 1 × 10^6^ cells per mL before adding empty NPs, dye-NPs, or dye-VD3-NPs to an end concentration of 200 μg/mL of the NPs. Blood samples were incubated for 15 min or 2 h at 4 °C or 37 °C. After incubation, the samples were washed with cold PBS to remove excess NPs, after which they were resuspended in 5–10 mL ACK lysis buffer for 10 min at 4 °C. The cells were then washed with PBS. The uptake of NPs by various immune cells was analysed by flow cytometry.

### 2.5 Cell isolation

Monocytes and B cells were isolated from buffy coats of healthy donors (Bloodbank Sanquin, Nijmegen). First, peripheral blood mononuclear cells (PBMCs) were isolated by Lymphoprep (18061, Serumwerk Bernburg) density gradient centrifugation. Monocytes and B cells were then isolated using human CD14 and CD19 MicroBeads (130-050-201 and 130-050-301, Miltenyi Biotec) respectively, according to manufacturer's protocol. After isolation, the cells were suspended in X-VIVO medium containing 2 % human serum (HS) (H4522, Merck).

Neutrophils were isolated from heparin-treated blood from healthy donors. The blood was first diluted 1:1 with PBS after which serum and PBMCs were removed by Lymphoprep density gradient centrifugation. Polynuclear cells were then collected and resuspended in 50 mL ACK lysis buffer for 10 min at 4 °C. The cells were centrifuged at 1500 rpm for 8 min at 10 °C and subsequently resuspended in 10 mL ACK lysis buffer for 5 min at 4 °C. The cells were then washed with PBS and resuspended in Iscove's Modified Dulbecco's Medium (IMDM) (124453, Gibco) containing 10 % Fetal Bovine Serum (FBS) (SV30160.03, HyClone).

### 2.6 B cell, neutrophil and monocyte treatment

For all cellular experiments, cells were left untreated or were treated with VD3-NPs, soluble VD3, or empty NPs (VD3 treatment) at the timepoints described below. VD3-NPs and soluble VD3 were added to a final concentration of 0.1 μM VD3, and the concentration of empty NPs was adjusted to the concentration of NPs added for VD3-NP conditions.

B cells were suspended at 1 × 10^6^ cells/mL in X-VIVO medium containing 2 % HS and plated with 200.000 cells/well in a 96-well U-bottom plate. The cells were stimulated with IL-21 (250 ng/mL) (200-21, Peprotech) and CD40L (2 μg/mL) (591706, BioLegend) in addition to VD3 treatment as described above. The cells were incubated at 37 °C for 9 days, after which the phenotype was assessed by flow cytometry analysis.

Neutrophils were resuspended at 1 × 10^6^ cells/mL in IMDM medium containing 10 % FBS and plated with 100.000 cells/well in a 96-well F-bottom plate. The cells were stimulated with LPS and VD3 treatment as described above and incubated at 37 °C for 2 h after which the phenotype was assessed by flow cytometry analysis. To measure IL-8 secretion by neutrophils, the cells were incubated for 24 h at 37 °C, after which supernatant was harvested.

Monocytes were resuspended at 1 × 10^6^ cells/mL in X-VIVO medium containing 2 % HS and plated at 100.000 cells/well in a 96-well U bottom plate. The cells were treated with VD3 stimuli as described above followed by 24 h incubation at 37 °C. Cells were then stimulated with 100 ng/mL LPS and incubated for 24 h at 37 °C, after which supernatant was harvested, and the phenotype was assessed using flow cytometry analysis.

### 2.7 Flow cytometry

Following cell treatment, cells were collected, washed with PBS, and stained with 1:2000 Zombie Violet (B cells, neutrophils, monocytes) (423114, BioLegend) or 1:1000 eFluor780 (whole blood) (65-0865-18, Invitrogen) viability dye in PBS for 20 min at 4 °C. The cells were washed with PBA and stained with a conjugated antibody mix in PBA for 20 min at 4 °C. For the viability of whole blood after overnight treatment with VD3-NP or soluble VD3, the cells were stained using conjugated antibody CD45 PE (1:50, 304008, BioLegend) (Supplementary Figure 1A). To assess the uptake of dye-NPs in whole blood, the cells were stained with an antibody mix containing CD15 PerCP-Cy5.5 (1:20, 560828, BD Biosciences), CD16 PerCP-Cy5.5 (1:20, 560717, BD Biosciences), CD14 PE-Cy7 (1:50, 557742, BD Biosciences), CD20 FITC (1:20, 345792, BD Biosciences), CD3 BV421 (1:50, 344834, BioLegend) and CD45 PE (1:50) **(**Supplementary Figure 1B**)**. The neutrophils were stained with an antibody mix containing CD66b FITC (1:50, 555724, BD Biosciences), CD15 PerCP-Cy5.5 (1:50), CD62L PE (1:100, 555544, BD Biosciences), CD16 APC-Cy7 (1:100, 302018, BioLegend), CD63 APC (1:50, 353008, BioLegend) **(**Supplementary Figure 2A). The B cells were stained with an antibody mix containing CD27 PE (1:50, 302808, BioLegend), CD20 BV510 (1:50, 302340, BioLegend), HLA-DR PerCP (1:40, 307628, BioLegend), CD80 PE-Cy7 (1:40, 561135, BD Biosciences), CD19 APC (1:40, 17-0199-42, eBioscience) and CD38 APC-H7 (1:50, 656646, BD Biosciences) (Supplementary Figure 2B). The monocytes were stained with an antibody mix containing CD86 PE (1:50, 555658, BD Biosciences), CD14 APC-H7 (1:50, 560180, BD Biosciences), MERTK PE-Cy7 (1:50, 367610, BioLegend), HLA-DR PerCP (1:40), CD11b APC (1:50, A87782, Beckman Coulter) (Supplementary Figure 2C). Following the incubation, the cells were washed with PBA and resuspended in PBA prior to flow cytometry analysis. Flow cytometry was performed on the BD FACSLyric (BD Biosciences) and analysed using FlowJo software (Version 10.10.0, BD Biosciences). Cell purities were confirmed for neutrophils (CD15^+^, >85 % for all donors) using CD15 PerCP-Cy5.5; for B cells (CD19^+^, >95 % for all donors) using CD19 APC; and for monocytes (CD14^+^, >95 % for all donors) using CD14 APC (1:50, 301808, BioLegend) on the day of isolation (Supplementary Figure 2).

### 2.8 Cytokine and immunoglobulin detection

For cytokine detection, supernatant of treated cell cultures was harvested and stored at −80 °C (whole blood) or −20 °C (B cells, neutrophils, monocytes). Cytokine or immunoglobulin (Ig) G concentrations were determined using human ELISA Kits detecting IL-6 (88-7066-88, ThermoFisher), IL-10 (88-7106-88, ThermoFisher), IL-23 (88-7237-88, ThermoFisher) and TNFα (88-7346-88, ThermoFisher) for whole blood and monocyte cultures; IL-8 (88-8086-88, ThermoFisher) for neutrophil cultures; and total IgG (88-50550-88, ThermoFisher) for B cell cultures, according to manufacturer's protocols.

### 2.9 Statistical analysis

Statistical analysis was performed with GraphPad Prism 10 (Version 10.1.1, GraphPad Software). Results are depicted as bar graphs showing mean ± SEM with individual values displayed as a scatter dot plot. Before statistical analysis, the various conditions were tested for normal distribution with the Anderson-Darling test. Significance between conditions was assessed by a matched one-way analysis of variance (ANOVA) followed by Bonferroni post-test for normally distributed data, or the Friedman test followed by Dunn's multiple comparisons test for non-normally distributed data. For normalized presented data, statistical analysis was performed on raw values prior to normalization. Statistical significance is depicted as follows: ∗p < 0.05, ∗∗p < 0.01, ∗∗∗p < 0.001. For non-significant comparisons, the p-values are depicted in graph.

## 3 Results

### 3.1 VD3-NPs inhibit cytokine secretion in whole blood

**Fig. 1:**
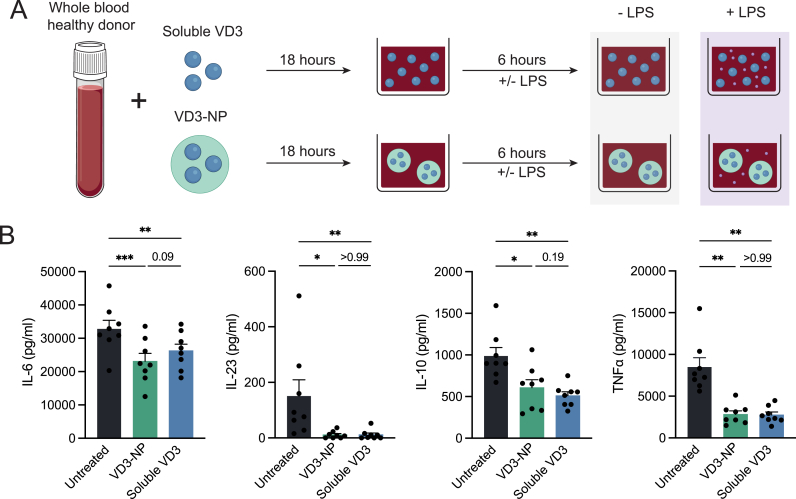
VD3-NPs inhibit cytokine secretion in whole blood. **(A)** Schematic overview of experimental setup. Whole blood from healthy donors was diluted 1:1 and treated with soluble VD3 or VD3-NP for 18 h, after which samples were stimulated with LPS to induce cytokine secretion. After 6 h of LPS stimulation, the supernatant was collected and cytokine concentrations of IL-6, IL-23, IL-10 and TNFα were determined by ELISA. **(B)** IL-6, IL-23, IL-10 and TNFα levels (pg/ml) in LPS-stimulated samples. Bar graphs depict mean ± SEM and each data point represents a healthy donor (n = 8) from two independent experiments. (∗P > 0.05; ∗∗P > 0.01; ∗∗∗P > 0.001).

As initial proof of principle for the clinical implications of IV injection, VD3-NPs were challenged to attenuate inflammation in a whole blood experiment. To address the VD3 specific modulation, empty NPs were included as a control (Supplementary Table 1). To this end, diluted whole blood was treated with 0.1 μM VD3-NP, soluble VD3 or empty NPs for 18 h, in the presence of LPS for the last 6 h of culture (Fig. 1A). Both encapsulated and soluble VD3 similarly inhibited the secretion of pro-inflammatory cytokines IL-6, IL-23, and TNFα, as well as the anti-inflammatory cytokine IL-10, upon LPS stimulation (Fig. 1B). Interestingly, empty NPs induced a modest reduction in IL-6 secretion compared to the untreated control (Supplementary Figure 3A). However, the VD3-NPs exhibited a significantly stronger inhibitory effect over empty NPs, indicating that this enhanced suppression could be attributed to the encapsulated VD3. Lastly, flow cytometry analysis revealed no significant differences on viability of CD45^+^ cells following VD3 treatment (Supplementary Figure 3B). These results illustrate that both VD3-NP and soluble VD3 suppress the secretion of cytokines by immune cells in human whole blood.

### 3.2 Polymeric NPs are taken up by phagocytic immune cells

**Fig. 2:**
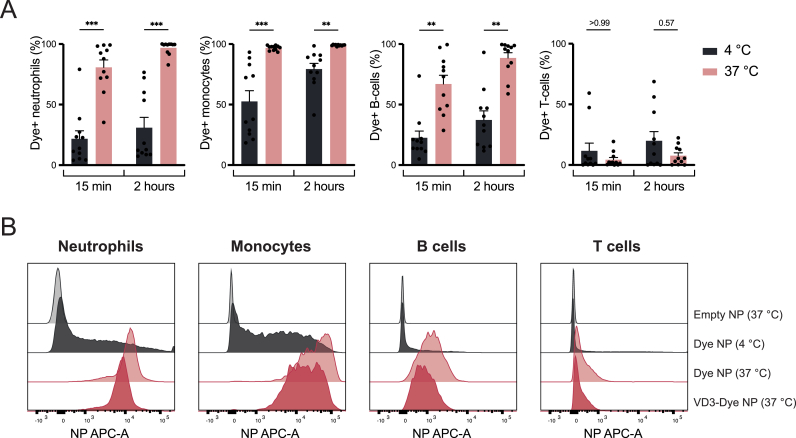
Polymeric NPs are taken up by phagocytic immune cells. Fluorescently labeled NPs without or with VD3 loading (Dye NP and VD3-Dye NP, respectively) were added to whole blood cultures at 4 °C or 37 °C for 15 min or 2 h. **(A)** NP uptake displayed as percentage of dye NP positive neutrophils, monocytes, B cells and T cells after stimulation for 15 min or 2 h at 4 °C and 37 °C. **(B)** Representative histograms displaying uptake of empty NPs (light gray), dye NPs at 4 °C (dark gray) and VD3-dye NPs at 4 °C (light red) or 37 °C (dark red). Bar graphs depict the mean ± SEM and each data point represents a healthy donor (n = 11) from three independent experiments. (∗P > 0.05; ∗∗P > 0.01; ∗∗∗P > 0.001).

Given the immunosuppressive effect observed in whole blood, we aimed to identify which immune cells are primarily modulated by the VD3-NPs. To this end, we characterized which immune cells most prevalently present in whole blood (neutrophils, monocytes, B cells and T cells) are actively taking up the NPs. Therefore, fluorescently labeled NPs (dye NPs) were added to whole blood for either 15 min or 2 h and NP uptake was analysed by flow cytometry. Active uptake was determined by comparing the number of dye-positive cells at 37 °C compared to those detected at 4 °C (indicative of non-active NP uptake or binding). Dye NPs were rapidly taken up by neutrophils, monocytes, and B cells as early as 15 min after treatment, while no uptake by T cells was observed (Fig. 2A). Furthermore, we examined the impact of NP loading with VD3 on cellular uptake. Hence, fluorescently labeled NPs, either loaded with VD3 (VD3-Dye NPs) or without VD3 (dye NPs) were added to whole blood at 37 °C for 2 h. VD3 loading did not significantly impact NP uptake, resulting in comparable uptake patterns across all characterized immune cell subsets (Fig. 2B, Supplementary Figure 4). While differences in gMFI signal could be observed, suggesting potential variations in the amount of NP internalized per cell, the proportion of NP-positive cells remained consistent across conditions. In the context of this study, we report that VD3 loading does not alter the overall frequency of immune cells taking up NPs. Together, these findings indicate that polymeric NPs are rapidly and actively taken up by phagocytic peripheral blood immune cells, namely neutrophils, monocytes, and B cells.

### 3.3 VD3-NPs alter IL-8 secretion by neutrophils

**Fig. 3:**
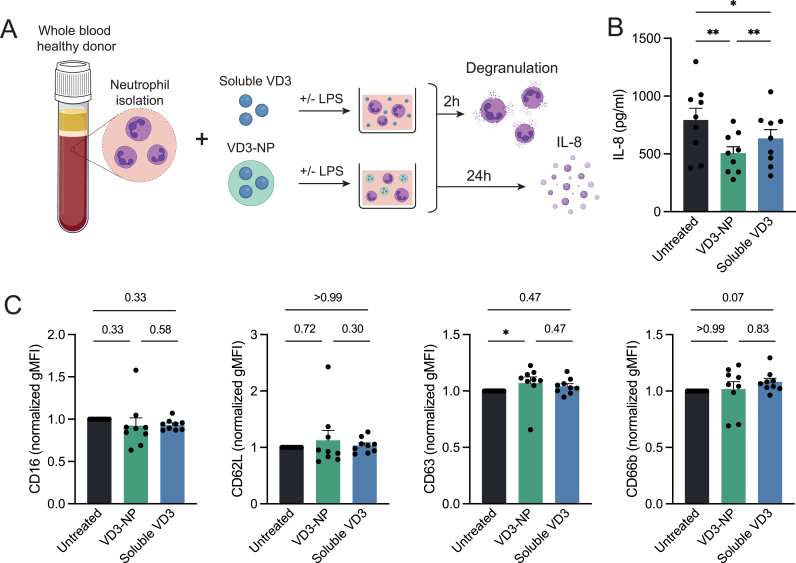
VD3-NPs alter IL-8 secretion by neutrophils. **(A)** Schematic of the experimental setup. Neutrophils were isolated from whole blood from healthy donors and treated with soluble VD3 or VD3-NP in combination with LPS for 2 h to determine degranulation status, or 24 h to determine IL-8 secretion. **(B)** IL-8 levels (pg/ml) in LPS-stimulated samples **(C)** CD16, CD62L, CD63, CD66b surface expression levels in LPS-stimulated neutrophils depicted in geometric mean fluorescent intensity (gMFI) normalized to the untreated samples. Bar graphs depict mean ± SEM and each data point represents a healthy donor (n = 9) from two independent experiments. (∗P > 0.05).

Given the prominent NP uptake by neutrophils, we aimed to determine the impact of VD3-NPs on neutrophil phenotype and function. Accordingly, neutrophils were treated with soluble VD3 or VD3-NPs followed by an LPS rechallenge (Fig. 3A). Neutrophils treated with empty NPs in combination with LPS showed a reduction in the pro-inflammatory mediator IL-8 compared to those treated with LPS alone, suggesting a baseline NP-related effect **(**Supplementary Figure 5A). However, VD3-NP treated neutrophils showed a more pronounced inhibition compared to LPS treated cells, underlining the VD3-specific effect on neutrophils (Fig. 3B, Supplementary Figure 5A). Moreover, no VD3 benefit in this regard was observed in the absence of LPS as IL-8 had not been induced. **(**Supplementary Figure 5A). Furthermore, we investigated the effect of VD3 and VD3-NPs on neutrophil phenotype and, particularly, on degranulation and viability. To this end, we characterized the expression of CD16, CD62L, CD63 and CD66b by flow cytometry. CD16 is cleaved from the neutrophil cell surface upon the release of secretory vesicles, leading to a decreased surface expression during degranulation [23]. CD62L, which facilitates neutrophil adhesion, is also downregulated upon activation [24]. In contrast, CD63 and CD66b are localized on granules that fuse to the cell membrane during degranulation, subsequently resulting in increased surface expression in degranulating neutrophils [25]. Of note, treatment with VD3 did not impact cell viability after 2 h of incubation (Supplementary Figure 5B). As expected, LPS stimulation led to a strong activated phenotype and degranulation in neutrophils, indicated by the reduced expression of CD16 and CD62L and increased expression of CD63 and CD66b (Supplementary Figure 5C). Neither soluble VD3 or VD3-NP treatment affected LPS-induced activation and degranulation (Fig. 3C). However, we noted a modest upregulation in CD63, hinting towards a more activated neutrophil phenotype. In conclusion, while VD3-NP treatments were shown to inhibit LPS-induced IL-8 secretion by neutrophils, it did not alter LPS-induced degranulation.

### 3.4 VD3-NPs shape B cell differentiation and antibody secretion

**Fig. 4:**
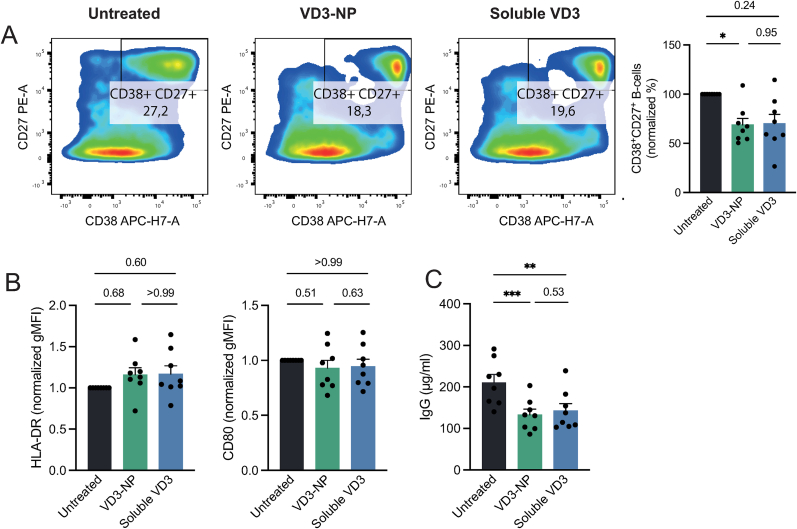
VD3-NPs shape B cell differentiation and antibody secretion. CD19^+^ B cells were isolated from buffy coats from healthy donors and treated with soluble VD3 or VD3-NP in combination with CD40L and IL-21. After 9 days, surface marker expression was determined by flow cytometry and supernatant was harvested for analysis of antibody secretion. **(A)** Representative dot plots showing expression levels of CD38 and CD27 on B cells together with a bar graph depicting the percentage of CD38^+^CD27^+^ double positive B cells, normalized to the untreated condition. **(B)** CD80 and HLA-DR expression on CD19^+^ B cells depicted in gMFI normalized to the untreated conditions. **(C)** Immunoglobulin G (IgG) levels (μg/ml) in culture supernatant. Bar graphs depict the mean ± SEM and each data point represents a healthy donor (n = 8) from two independent experiments. (∗P > 0.05; ∗∗P > 0.01; ∗∗∗P > 0.001).

Autoimmunity is driven by the production of autoantibodies by plasma cells, contributing to tissue damage and inflammation [2]. To explore whether soluble VD3 or VD3-NPs impact B cell differentiation into antibody-secreting plasma-like cells, CD19^+^ isolated B cells were treated with CD40-ligand (CD40L) and IL-21 together with soluble VD3 or VD3-NP for 9 days. B cell differentiation into plasma-like cells was determined by the co-expression of CD38 and CD27 by flow cytometry. Noteworthy, we observed that all treatments improved B cell viability after 9 days of culture (Supplementary Figure 6A). Moreover, VD3-NP treatment significantly reduced the abundance of plasma-like B cells compared to empty NPs, indicated by the lower percentage of CD38^+^CD27^+^ B cells (Fig. 4A, Supplementary Figure 6B). In contrast, the expression of the co-stimulatory molecule CD80 or the antigen-presenting molecule HLA-DR remained unchanged following treatment with VD3 or empty NPs (Fig. 4B, Supplementary Figure 6C). To determine whether the reduced ratio of plasma-like cells correlated with a functional suppression of antibody production, the culture supernatants were tested for total IgG levels. Both VD3-NP and soluble VD3 treatment strongly decreased IgG secretion, whereas empty NP treatment resulted in a slight, non-significant reduction of IgG (Fig. 4C, Supplementary Fig. 6D). Together, these results demonstrate that VD3-NPs suppress B cell differentiation into CD38^+^CD27^+^ cells and robustly suppress the antibody production.

### 3.5 VD3-NPs attenuate pro-inflammatory responses in monocytes

**Fig. 5:**
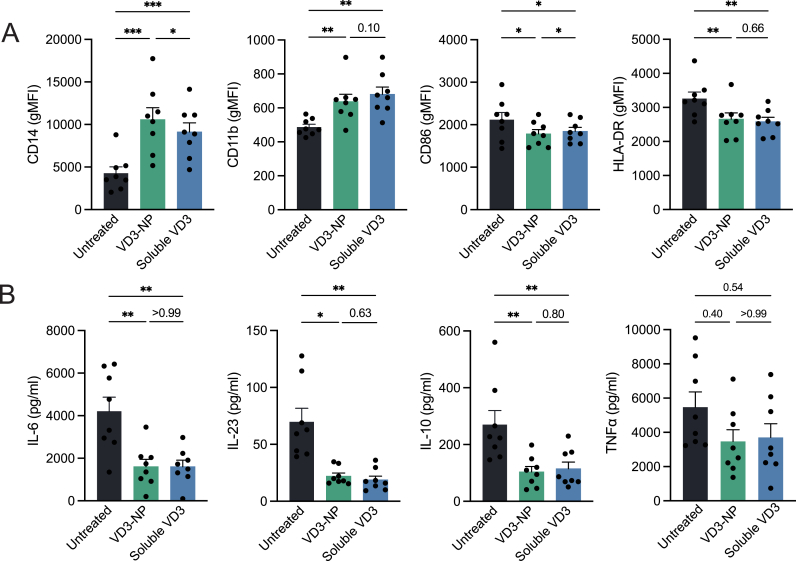
VD3-NPs attenuate inflammation in monocytes. Monocytes were isolated from buffy coats and treated with soluble VD3 or VD3-NP for 24 h, after which samples were stimulated with LPS for another 24 h. Following incubation, marker expression was analysed using flow cytometry and cytokine levels in the supernatant were determined by ELISA. **(A)** CD14, CD86, CD11b, and HLA-DR surface expression levels in LPS-stimulated monocytes depicted in bar graphs displaying gMFI. **(B)** IL-6, IL-23, IL-10 and TNFα levels (pg/ml) in LPS-stimulated samples. Bar graphs depict mean ± SEM and each data point represents a healthy donor (n = 8) from two independent experiments. (∗P > 0.05; ∗∗P > 0.01; ∗∗∗P > 0.001).

Given their prominent role as antigen-presenting cells (APCs) in the peripheral blood, monocytes represent a relevant target for investigating the immunomodulatory potential of VD3 to combat autoimmunity. To this end, CD14^+^ monocytes were isolated from buffy coats derived from healthy donors and treated with soluble VD3 or VD3-NPs for 24 h, after which the monocytes were rechallenged with LPS for an additional 24 h. Following treatment, surface marker expression was determined using flow cytometry. Importantly, VD3 treatment did not affect overall monocyte viability (Supplementary Figure 7A). We observed VD3 to downregulate the maturation markers, CD86 and HLA-DR, while mediating the upregulation of the tolerogenic markers CD11b and CD14 (Fig. 5A). Additionally, we observed these differences in monocytes regardless of the LPS rechallenge (Supplementary Figure 7B). MERTK, a receptor often expressed on suppressive myeloid cell types, was unexpectedly downregulated upon empty NP treatment, but showed minimal, non-significant changes upon VD3 treatment. Lastly, empty NP treatment increased CD11b expression in non-LPS stimulated samples, however, this was significantly higher in VD3-NP treated samples, demonstrating the benefit of VD3 loading on the NP (Supplementary Figure 7B). To investigate whether VD3 treatment affects monocyte function, we analysed cytokine secretion in the supernatant. In absence of LPS rechallenge, monocytes produced negligible amounts of cytokines, however, with LPS rechallenge, VD3 treatment strongly dampened the secretion of pro-inflammatory cytokines IL-6 and IL-23, and anti-inflammatory cytokine IL-10. As for TNFα, we noted a tendency for most donors to also exhibit a reduced TNFα secretion upon VD3 treatment, although not significant (Fig. 5B, Supplementary Figure 8). These data illustrate the profound impact of VD3-NPs on monocytes to attenuate their pro-inflammatory functions while priming a suppressive phenotype.

## 4 Discussion

VD3 has been widely recognized for its immunomodulatory properties, and much effort has been drawn to harness the therapeutic value of soluble or encapsulated VD3 for the induction of immune tolerance and attenuation of hyperactive immunity in autoimmune diseases [[18], [19], [20],[26], [27], [28], [29]]. However, mounting clinical interventions with VD3 have demonstrated mixed responses in humans, arguing for the need to improve the efficacy of VD3-based therapies [30,31]. Given the potential toxic side effects and inherent instability of the active metabolite of VD3, it is worthwhile to explore alternative delivery methods to improve *in vivo* efficacy and safety. Here, we present a proof-of-principle of a PLGA nanoparticle-based VD3 delivery system to target the human phagocytic system and attenuate pro-inflammatory responses.

Hinting towards a potential IV route NP application, we sought to address the effect of the NPs in a whole blood experiment. Here, we observed that treatment with VD3-NPs suppressed the secretion of the pro-inflammatory cytokines IL-6, IL-23, and TNFα to an extent comparable to that of soluble VD3. This is consistent with previous work demonstrating similar pro-inflammatory cytokine modulation of VD3 in human whole blood and PBMC-based assays [[32], [33], [34], [35]]. Notably, we observed a reduction in IL-10 following VD3 treatment, regardless of LPS rechallenge. Literature describes a dual role for VD3 in the regulation of IL-10, with both upregulation [32,33,35] and downregulation [[36], [37], [38], [39], [40], [41]] reported, suggesting a pleiotropic effect. This variability is hypothesised to be influenced by factors such as the specific cell types targeted and the duration of exposure to VD3. Interestingly, short-term exposure of monocytes to VD3 has been associated to a reduced IL-10 secretion, whereas prolonged exposure has been shown to promote IL-10 secretion [41]. We hypothesize that VD3 in our setup induces immunosuppression through broad suppression of inflammatory pathways, including both pro- and anti-inflammatory cytokines, as opposed to the induction of IL-10 specifically.

In depth characterization of immune subsets targeted by the NPs showed that these are preferentially taken up by professional phagocytes in blood (monocytes, neutrophils and B cells). Importantly, we noted no uptake by T cells, indicating these PLGA NPs to be exclusively targeted to the phagocytic compartment of the peripheral immune system. Of note, the presence of VD3 loading did not impair NP uptake.

To further dissect the cell-specific effects of VD3, we examined cytokine responses and phenotypic changes across the isolated immune cell subsets that actively take up the NPs. In neutrophils, VD3-NPs significantly suppressed the LPS-induced secretion of IL-8, a key chemokine involved in neutrophil activation and migration [42]. While this observation aligns with prior studies using similar LPS rechallenge models [43,44], it contrasts with other reports suggesting that VD3 either has no effect or may enhance IL-8 release [45,46]. A potential explanation for this difference may lie in different activation stimuli or treatment dosage. As for the monocytes, we noted a robust inhibition in all cytokine secretion upon VD3 treatment, aligning with our prior results in whole blood data and existing studies conducted in monocytes [39,[47], [48], [49]]. Notably, VD3 has been shown to downregulate Toll-like receptor (TLR) expression on monocytes [48,49], resulting in decreased response upon to a TLR rechallenge, which may contribute to this effect.

Phenotypically, we did not observe differences in neutrophils upon treatment with VD3, aligning with prior reports showing no impact in degranulation and neutrophil activation [45]. This mechanism allows VD3 to dampen pro-inflammatory cytokine secretion without impairing essential effector functions, highlighting its potential as a therapeutic modulator. In contrast, the VD3-NPs exhibited a profound effect on monocyte phenotype. VD3 treated monocytes showed an increased expression of CD14 and CD11b, markers often upregulated on suppressive or anti-inflammatory myeloid subsets, along with a reduction of HLA-DR and CD86, key molecules involved in antigen presentation and co-stimulation [50]. Collectively, these results indicate that VD3-NPs can successfully attenuate inflammatory responses across major innate immune cells while priming, at least on monocytes, an immunosuppressive phenotype.

Existing literature has indicated the suppressive effect of VD3 over B cell differentiation into plasma B cells as well as on IgG production, both critical aspects for the development of a humoral immune response [[51], [52], [53], [54]]. To mimic plasma B cell differentiation *in vitro*, we primed isolated B cells with CD40L and IL-21 together with VD3. Here, we observed that VD3-NP treatment reduced the *in vitro* differentiation of B cells into CD27^+^CD38^+^ (plasma like) cells whereas no differences were observed in HLA-DR or CD80 expression. More importantly, we observed a robust impairment in IgG production upon VD3 treatment. Although our result contrast with one prior report showing the negative role of VD3 on B cell co-stimulatory markers expression [55], they are consistent with the notion that VD3 limits plasma cell differentiation [51] and antibody production [[51], [52], [53], [54]]. Together, our results indicate that VD3 encapsulated in NPs possess a similar, possibly even stronger, immune modulating effect on B cells, compared to soluble VD3.

Interestingly, across multiple experiments we observed immunomodulatory effects upon treatment with empty NPs. This was evidenced by a general reduction in cytokine production and an altered monocyte phenotype. Although we observed that the PLGA formulation may inherently already suppress inflammatory responses, the encapsulated VD3 significantly amplified this effect. Aligning with these reported immunomodulatory effects, prior reports have indicated PLGA formulations to vary in their immunogenicity depending on factors such as particle size, charge, dosage, and timing [[56], [57], [58], [59], [60], [61], [62]]. The notion that stimulation with PLGA-based particles may dampen the immune response has been described before, an effect hypothesised to arise as an off-target effect caused by the intracellular degradation of the polymer [58,61,63]. Adding up to these studies, we provide evidence that our generated PLGA NPs display a rather anti-inflammatory effect, that in synergy with VD3, represents an attractive platform for the attenuation of inflammation and the skewing towards tolerogenic immunity.

NP encapsulation offers advantages over soluble drug formulations, including protection from degradation and possibility of co-delivery with disease-specific antigens. Having established the efficacy of encapsulated VD3, further studies should be focused on investigating the incorporation of disease-relevant antigens into the NP formulation, aiming to drive antigen-specific immune tolerance in autoimmune disorders. Additionally, PLGA-based NPs in particular offer further benefits due to their biodegradability and established regulatory approval for use in humans. We propose IV administration of PLGA NPs, as this has proven successful immunogenicity in preclinical studies [64], and is currently being evaluated in a phase I clinical trial (NCT04751786) [65].

In summary, we demonstrated that VD3-NPs effectively suppress the major phagocytic subsets in human blood; namely monocytes, B cells, and neutrophils, hampering pro-inflammatory cytokine, chemokine and antibody production. These findings highlight VD3-NPs as a promising platform for the treatment of inflammation-driven or autoimmune disorders. Nonetheless, several limitations of this study should be acknowledged. A key limitation is the pleiotropic nature of VD3, as its immunomodulatory effects may depend on donor baseline VD3 levels and VDR polymorphisms, both of which have been shown to affect VD3 responsiveness [[66], [67], [68]]. Future studies should assess donor VD3 status and determine whether responsiveness is linked to baseline levels of VD3. Moreover, follow-up studies should address whether VD3 encapsulation alters biodistribution or toxicity of PLGA NPs *in vivo*. Another important limitation of this work is the lack of investigation into the effect of VD3-NPs on dendritic cells (DCs), which serve as professional antigen-presenting cells bridging innate and adaptive immunity. Given their importance in the induction of either pro-inflammatory or tolerogenic T cell immunity, it is crucial to examine how VD3-NPs affect human DC subsets and, subsequentially, their impact on T cell functions.

Taken together, our results validate the therapeutic potential of VD3 encapsulated in PLGA NPs, proven by their capacity to impair the development of pro-inflammatory immunity. This positions VD3-NPs as an attractive strategy for the treatment of autoimmune diseases.

## CRediT authorship contribution statement

**Julia Minnee:** Writing – original draft, Visualization, Methodology, Investigation, Formal analysis, Data curation, Conceptualization. **Jorge Cuenca-Escalona:** Writing – review & editing, Supervision, Methodology, Investigation, Conceptualization. **Johanna Bödder:** Writing – review & editing, Methodology, Conceptualization. **Georgina Flórez-Grau:** Writing – review & editing, Supervision, Methodology, Conceptualization. **Esther C. de Jong:** Writing – review & editing, Supervision, Methodology, Conceptualization. **I. Jolanda M. de Vries:** Writing – review & editing, Supervision, Funding acquisition, Conceptualization.

## Ethics

Blood tubes and buffy coats from healthy donors were acquired from Bloodbank Sanquin after written consent and according to institutional guidelines.

## Funding

This work was funded by Health∼Holland grant ImmuneHealthSeed (LSHM22042-SGF)

## Declaration of competing interest

Authors declare no conflict of interest.

## Data Availability

Data will be made available by the author upon reasonable request.
