# Supplementary material for: Vitamin D3 encapsulated in polymeric nanoparticles to dampen the pro-inflammatory immune response
Source: J Transl Autoimmun. 2025 Sep 30;11:100321. doi: 10.1016/j.jtauto.2025.100321 (PMC12529509; doi:10.1016/j.jtauto.2025.100321)
Supplement: Multimedia component 1 [file mmc1.pdf]

Supplementary Table 1: Nanoparticle characterization
Size (nm), polydispersity index and VD3 weight percentage (wt%) after lyophilization of the NP.

Nanoparticle	Size (nm)	Polydispersity Index	Wt% VD3
Empty NP	158,3 $\pm$ 46,6	0.06	n/a*
VD3-NP	175,4 $\pm$ 50	0.04	0.25
Dye-NP	226.8 $\pm$ 47	0.04	n/a*
VD3-Dye NP	216.7 $\pm$ 53	0.06	0.10

*N/a: not applicable

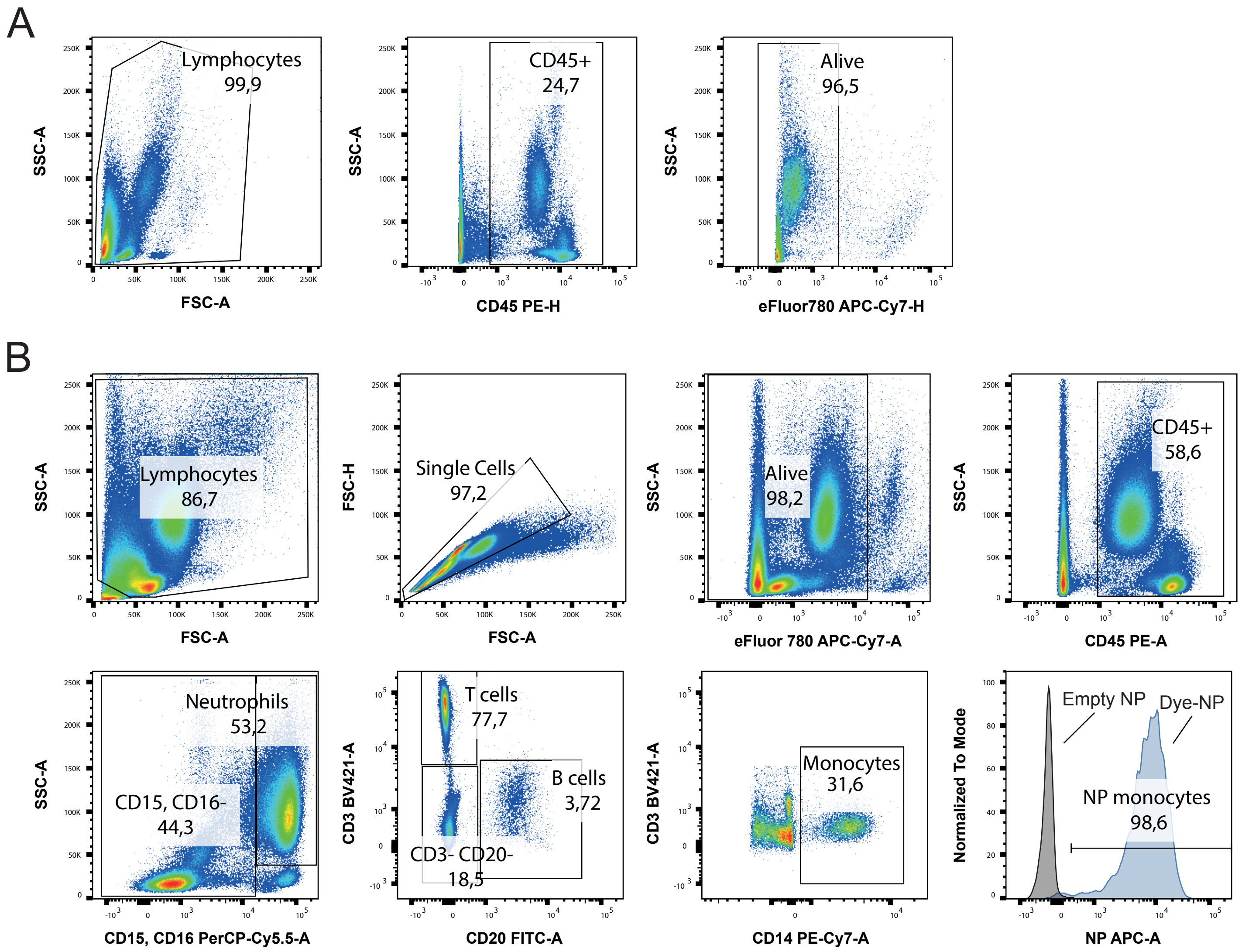

Supplementary Figure 1: Gating strategies whole blood

(A) Flow cytometry gating strategy to determine immune cell viability. The first gate is set based on SSC-A and FSC-A, followed by a second gate on immune cells (PE-CD45+), thereby gating out leftover debris and erythrocytes as a result from the ACK lysis. Lastly, living cells were gated (eFluor780 negative population). **(B)** Gating strategy to determine uptake of fluorescently labeled NPs by different immune cells. The first gate is set based on SSC-A and FSC-A, followed by a gate on single cells using FSC-A and FSC-H. Living cells were gated as the eFluor780 negative population, after which immune cells were gated as CD45 positive. From the CD45+ population, the neutrophils were characterized by expression of CD15 and CD16. Within CD15-CD16- cells, T-cells and B-cells were characterized by the expression of CD3 and CD20, respectively. Of the CD3-CD20- population, monocytes were characterized by the expression of CD14. Within each cell type, the dye-NP uptake was determined by setting the gate in the APC channel with control samples stimulated with empty (non-fluorescent) NPs.

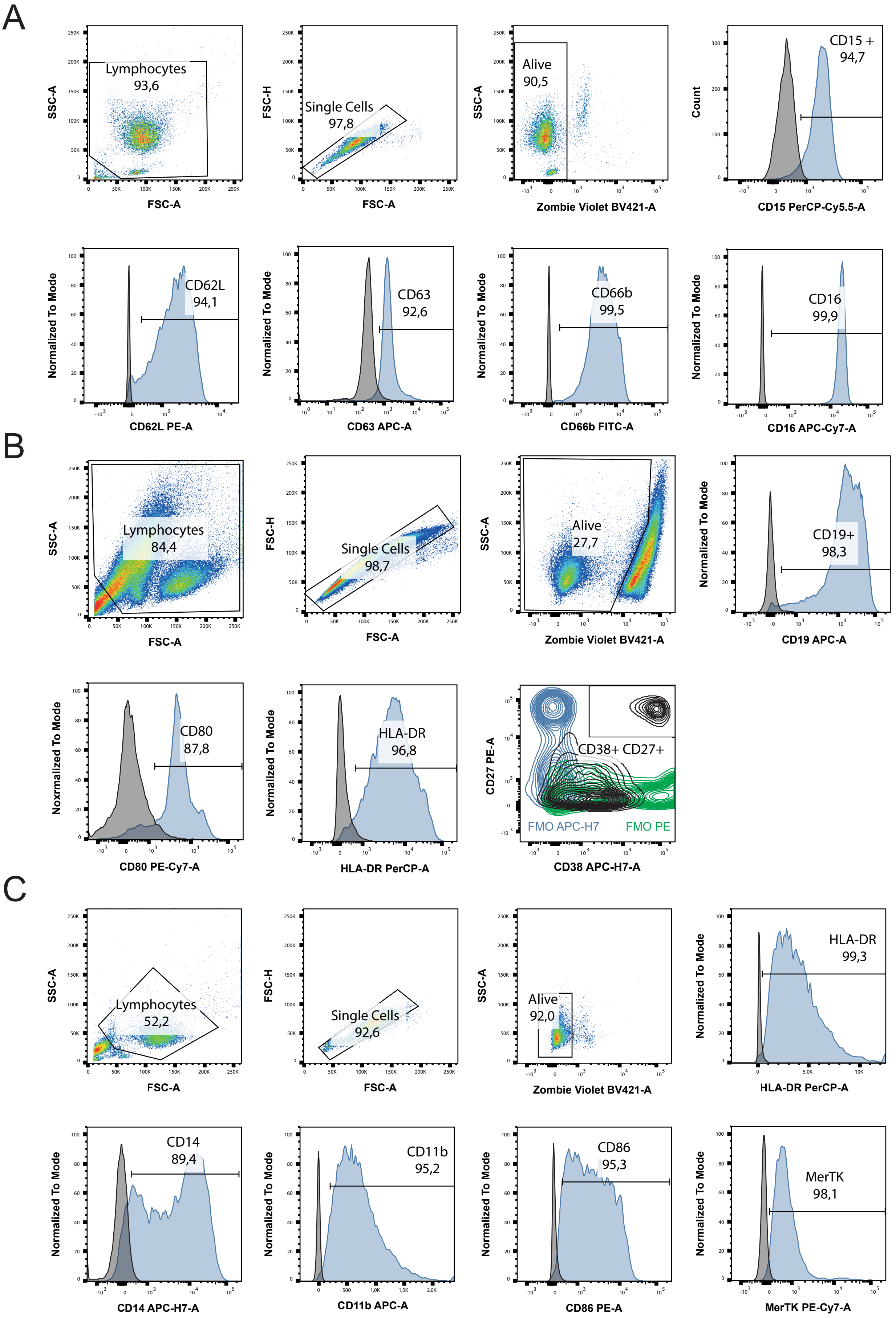

Supplementary Figure 2: Gating strategies isolated cells
Flow cytometry gating strategies to determine **(A)** neutrophil, **(B)** B cell, and **(C)** monocyte phenotype. First, cells were gated on SSC-A and FSC-A, followed by a single cell gate using FCS-A and FSC-H. Living cells were gated as the Zombie Violet negative population. The cell purity of neutrophils (CD15 PerCP-Cy5.5) and B cells (CD19 APC) are depicted in a histogram of a representative donor, on the day of isolation, with unstained samples in black and stained samples in blue. Marker expression at the end-point of the experiments for each cell type was determined by comparing stained samples (depicted in the blue histograms) with FMO controls (depicted in the grey histograms) for the indicated fluorochrome.

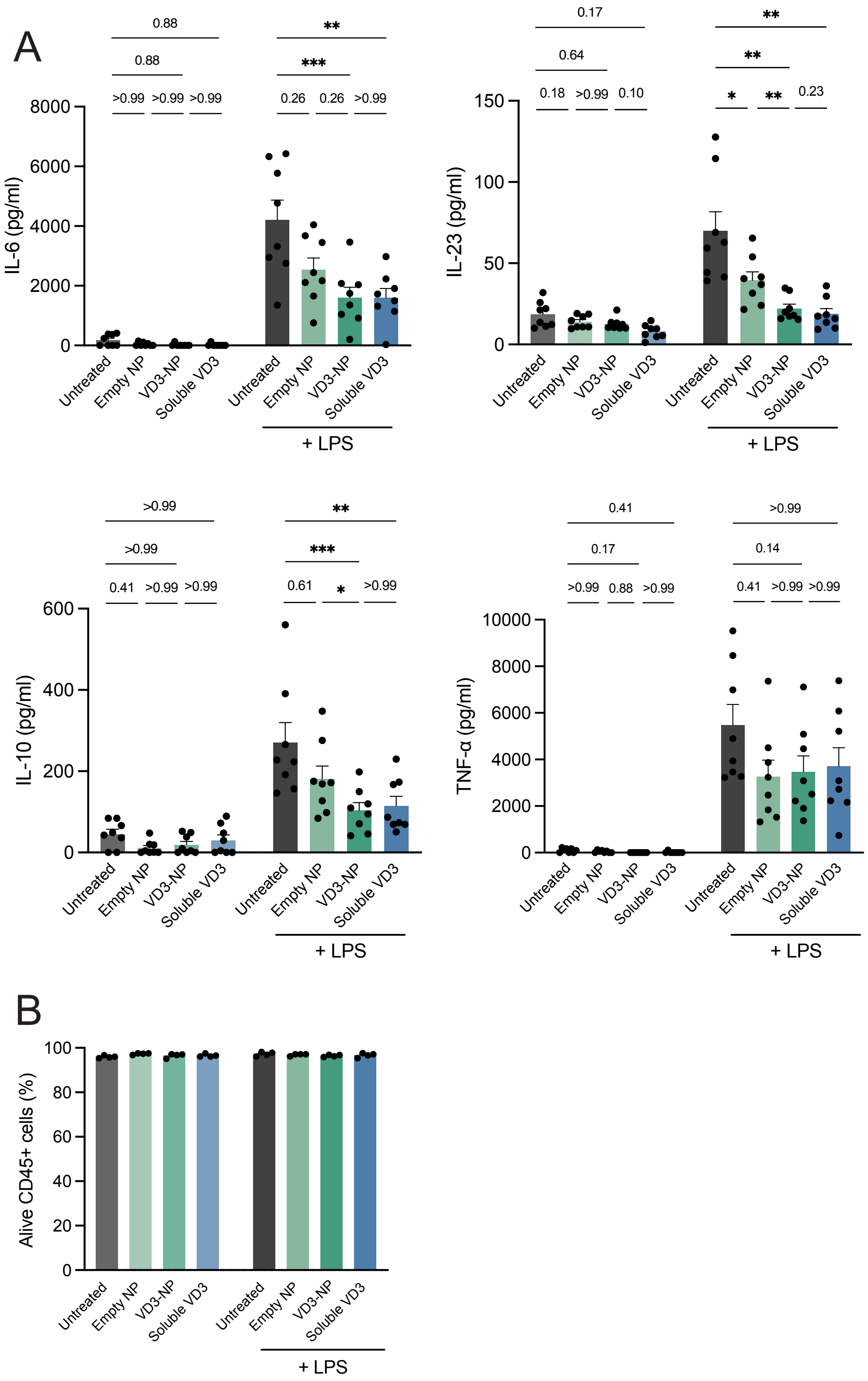

Supplementary Figure 3: Expanded whole blood data and viability

(A) IL-6, IL-23, IL-10 and TNF-α levels (pg/ml) in samples treated with VD3 or empty NPs, either left unstimulated or additionally rechallenged with 100ng/ml LPS. **(B)** Viability of immune cells in whole blood after treatment as described above, shown in percentage of living cells within the CD45+population. Bar graphs depict mean ±SEM and each data point represents a healthy donor (n=4, viability; n=8, cytokines) from one (viability) or two (cytokines) independent experiments. (*P>0.05; **P>0.01; ***P>0.001).

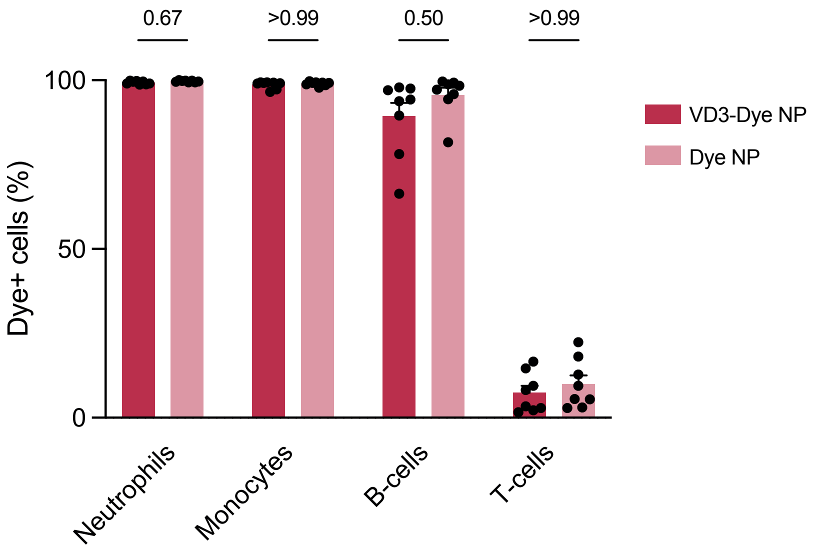

Supplementary Figure 4: VD3-NP uptake

NP uptake displayed as percentage of neutrophils, monocytes, B-cells and T-cells positive for fluorescent dye after stimulation with dye NPs with or without VD3 for 2 hours at 37 °C (VD3-Dye NP and Dye NP, respectively). Bar graphs depict the mean ±SEM and each data point represents a healthy donor (n=8) from two independent experiments.

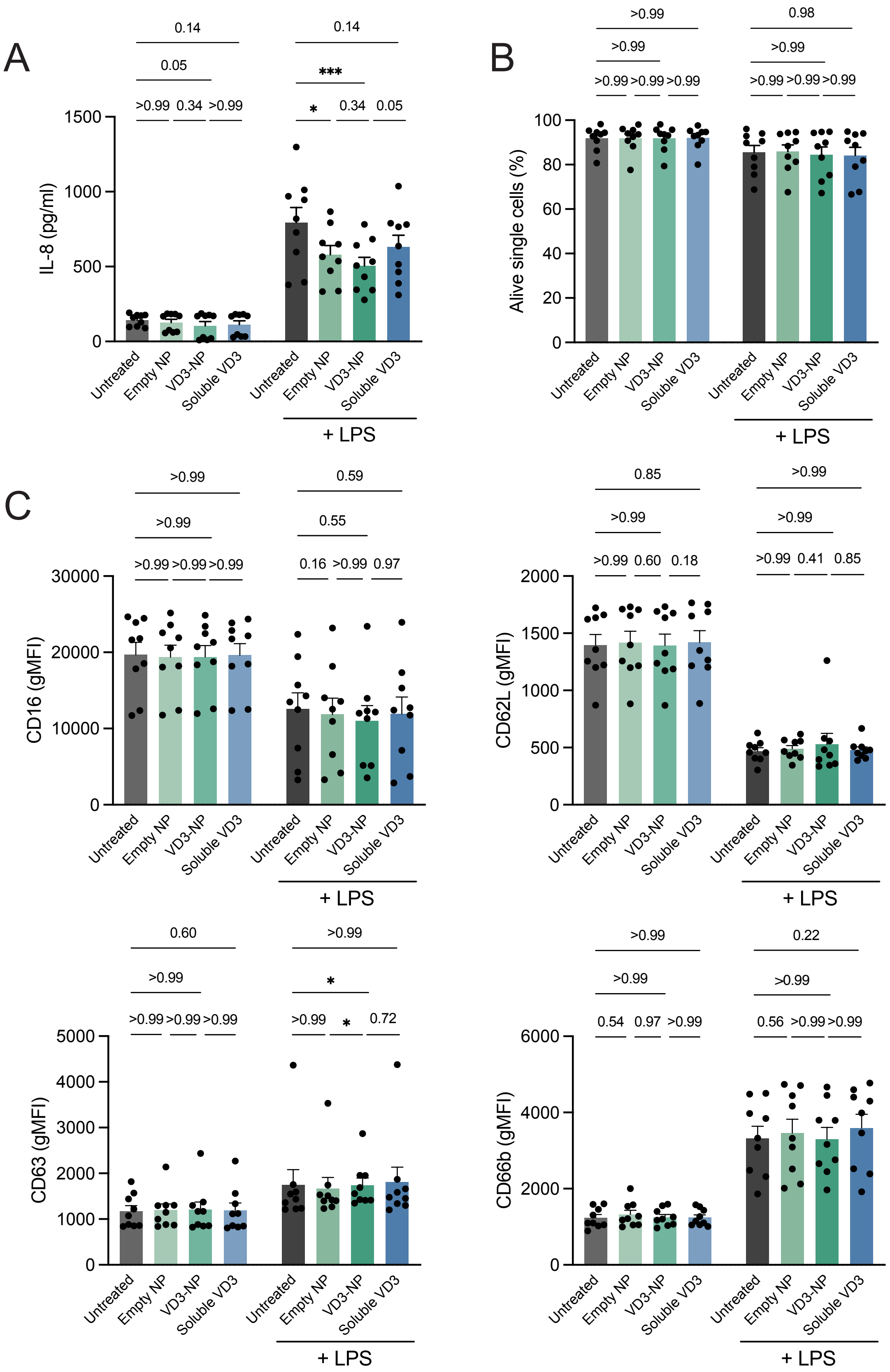

Supplementary Figure 5: Expanded neutrophil data and viability
(A) IL-8 levels (pg/ml) in supernatant of neutrophils treated with VD3 or empty NPs, either left unstimulated or additionally rechallenged with 100ng/mL LPS. **(B)** Single cell viability in percentages, and **(C)** CD16, CD62L, CD63, CD66b surface expression levels depicted in gMFI of neutrophils treated with VD3 or empty NPs, either left unstimulated or additionally rechallenged with 100ng/mL LPS. Bar graphs depict mean ±SEM and each data point represents a healthy donor (n=11) from two independent experiments. (*P>0.05; ***P>0.001)

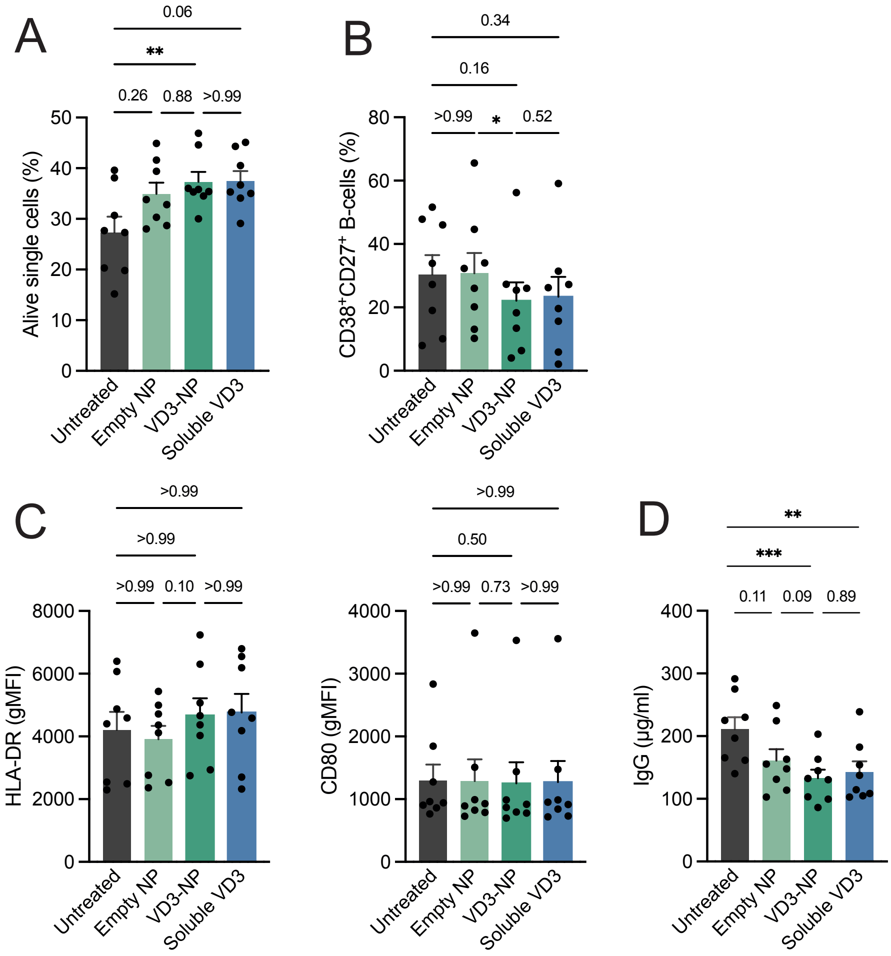

Supplementary Figure 6: Expanded B-cell data and viability

Bar graphs depicting **(A)** Viability in percentage of living cells, **(B)** percentage of $CD19^+CD38^+CD27^+$ double positive cells, **(C)** gMFI of HLA-DR and CD80 expression, and **(D)** Immunoglobulin G (IgG) levels (µg/ml) in culture supernatant after VD3 or empty NP treatment supplemented with CD40L and IL-21. Bar graphs depict the mean ±SEM and each data point represents a healthy donor (n=8) from two independent experiments. (*P>0.05; **P>0.01; ***P>0.001).

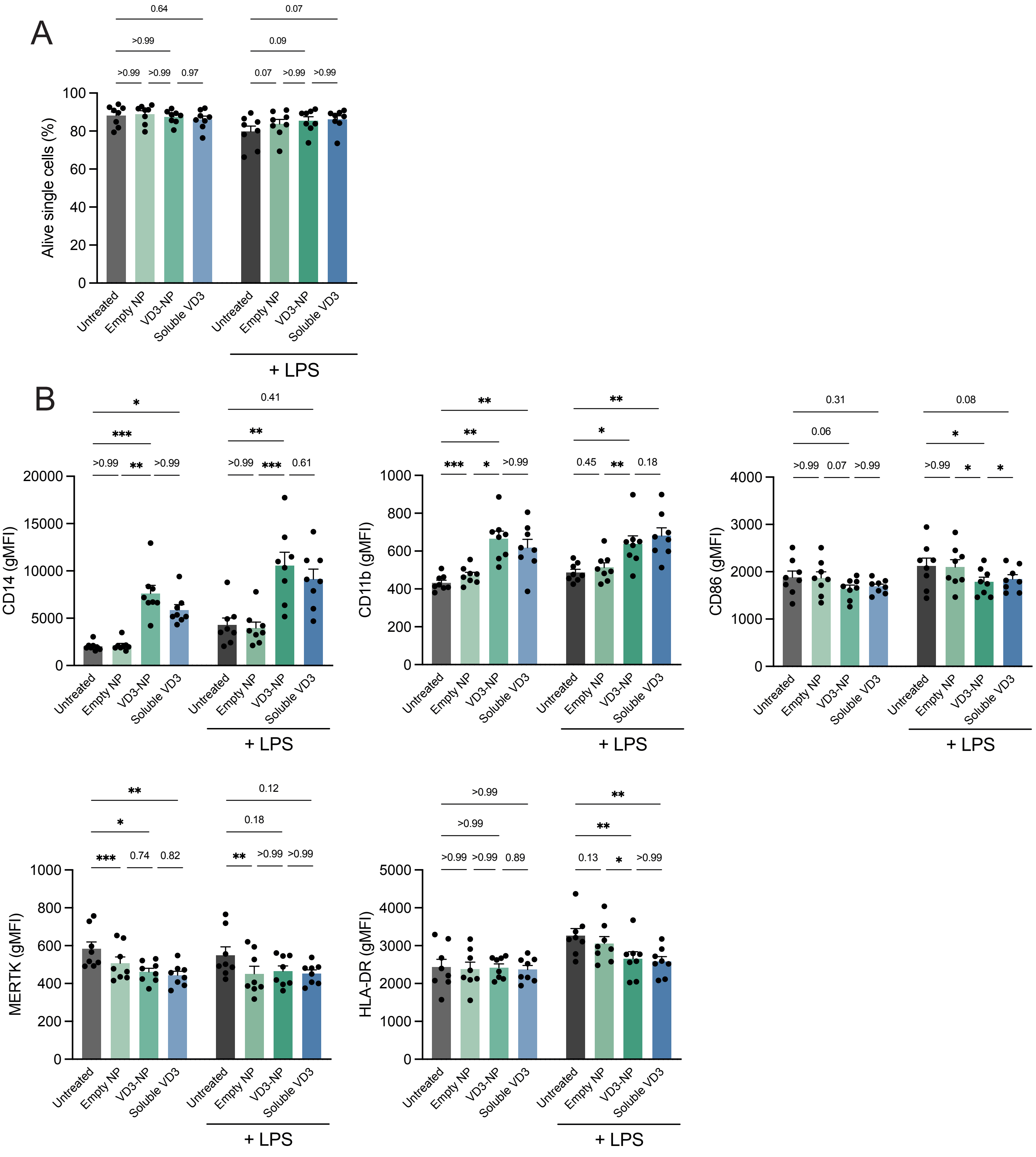

Supplementary Figure 7: Expanded monocyte flow cytometry data and viability

Bar graphs depicting **(A)** viability in percentage of living cells, **(B)** CD14, CD11b, CD86, MERTK and HLA-DR surface marker expression in gMFI of monocytes treated with VD3 or empty NPs, either left unstimulated or additionally rechallenged with 100ng/mL LPS. Bar graphs depict the mean ±SEM and each data point represents a healthy donor (n=8) from two independent experiments. (*P>0.05; **P>0.01; ***P>0.001).

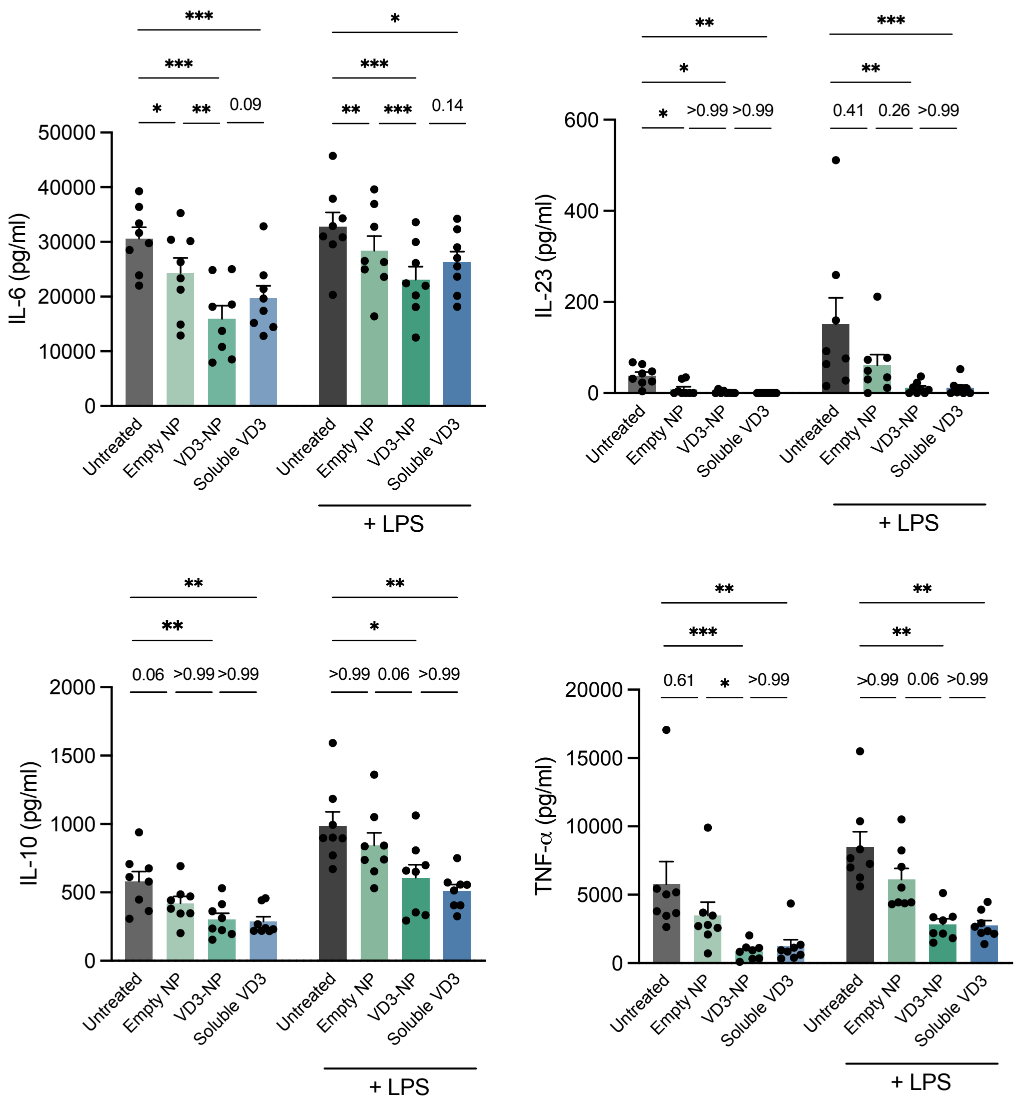

Supplementary Figure 8: Expanded monocyte cytokine data

Bar graphs depicting IL-6, IL-23, IL-10 and TNF-α levels (pg/ml) of monocytes treated with VD3 or empty NPs, either left unstimulated or additionally rechallenged with 100ng/mL LPS. Bar graphs depict the mean ±SEM and each data point represents a healthy donor (n=8) from two independent experiments. (*P>0.05; **P>0.01; ***P>0.001).
